# The Influence of a Conjugated Pneumococcal Vaccination on Plasma Antibody Levels against Oxidized Low-Density Lipoprotein in Metabolic Disease Patients: A Single-Arm Pilot Clinical Trial

**DOI:** 10.3390/antiox10010129

**Published:** 2021-01-18

**Authors:** Ronit Shiri-Sverdlov, Inês Magro dos Reis, Yvonne Oligschlaeger, Tim Hendrikx, Dennis M. Meesters, Annick Vanclooster, Nele Vanhoutvin, Ger H. Koek, Marit Westerterp, Christoph J. Binder, David Cassiman, Tom Houben

**Affiliations:** 1 Department of Molecular Genetics, School of Nutrition and Translational Research in Metabolism (NUTRIM), Maastricht University, 6229 ER Maastricht, The Netherlands; ines.reis@maastrichtuniversity.nl (I.M.d.R.); yvonneoligschlaeger@ziggo.nl (Y.O.); d.meesters@maastrichtuniversity.nl (D.M.M.); tom.houben@maastrichtuniversity.nl (T.H.); 2 Department of Laboratory Medicine, Medical University of Vienna, 1090 Vienna, Austria; tim.hendrikx@meduniwien.ac.at (T.H.); christoph.binder@meduniwien.ac.at (C.J.B.); 3 Liver Research Unit, University of Leuven, 3000 Leuven, Belgium; annick.vanclooster@uzleuven.be (A.V.); nele.vanhoutvin@uzleuven.be (N.V.); david.cassiman@uzleuven.be (D.C.); 4 Department of Gastroenterology-Hepatology and Metabolic Center, University Hospitals Leuven, 3000 Leuven, Belgium; 5 Internal Medicine, Division of Gastroenterology and Hepatology, Maastricht University Medical Centre, 6229 ER Maastricht, The Netherlands; gh.koek@mumc.nl; 6 Department of Pediatrics, University of Groningen, University Medical Center Groningen, 9700 RB Groningen, The Netherlands; m.westerterp@umcg.nl

**Keywords:** oxLDL, phosphorylcholine, pneumococcal immunization, anti-oxLDL antibodies, metabolic diseases

## Abstract

As a mediator between lipid metabolism dysfunction, oxidative stress and inflammation, oxidized low-density lipoprotein (oxLDL) is a promising therapeutical target in a wide range of metabolic diseases. In mice, pneumococcal immunization increases anti-phosphorylcholine and oxLDL antibody levels, and reduces atherosclerosis, non-alcoholic steatohepatitis and Niemann–Pick disease burden. These findings suggest that pneumococcal vaccination may be a useful preventive and therapeutical strategy in metabolic disease patients. In this pilot clinical trial, our aim was to determine whether the administration of a pneumococcal vaccine increases anti-phosphorylcholine and anti-oxLDL antibody levels in metabolic disease patients. The following patients were enrolled: four patients with familial partial lipodystrophy (all women, mean age 32 years old); three familial hypercholesterolemia patients (one girl, two boys; mean age 13 years); and two Niemann–Pick type B (NP-B) patients (two men, mean age 37.5 years old). Participants received one active dose of a 13-valent conjugated pneumococcal vaccine (Prevenar 13) and were followed-up for four weeks. Four weeks after Prevenar 13 vaccination, no differences were observed in patients’ levels of anti-oxLDL IgM or IgG antibodies. In addition, we observed a reduction in anti-phosphorylcholine (anti-PC) IgM antibody levels, whereas no differences were observed in anti-PC IgG antibody titers. These findings indicate that Prevenar 13 vaccination does not induce an immune response against oxLDL in patients with metabolic diseases. Therefore, Prevenar 13 is not suited to target the metabolic disruptor and pro-inflammatory mediator oxLDL in patients.

## 1. Introduction

Lipids perform extensive biological functions, including the modulation of cell membrane composition, acting as signaling molecules and contributing to the production and storage of energy. As such, lipid metabolism is a complex and tightly regulated whole-body process, and dysfunctions in lipid metabolism can lead to dire consequences for the organism. The importance of a properly functioning lipid metabolism is illustrated by the disease burden tied to the wide array of known acquired and inherited lipid metabolic diseases. Currently, widespread lifestyle habits, such as unhealthy diets and low physical activity, are major propellers of dyslipidemia and metabolic syndrome [1]. In addition, a considerable number of individuals are genetically predisposed to develop metabolic dysfunction, including familial hypercholesterolemia (FH), lipodystrophy (LPD) and Niemann–Pick (NP) disease patients. Regardless of the underlying etiology, patients with lipid metabolic dysfunctions are at high risk of developing non-communicable diseases such as type 2 diabetes, cardiovascular and liver disease and cancer, which are estimated to account for nearly 70% of premature deaths worldwide [2]. As such, the development of preventive and therapeutic strategies targeting disorders of lipid metabolism and associated disease burden is of utmost importance for patients, clinicians and societies alike.

Among other factors, an increase in the production of oxidized low-density lipoprotein (oxLDL) has emerged as an important metabolic disruptor and pro-inflammatory mediator in metabolic diseases and associated non-communicable diseases. OxLDL is broadly categorized as minimally or extensively modified LDL, and a wide range of oxLDL particles have been observed, varying both in chemical structure and biological effects [3]. Unlike native LDL, oxLDL binds to scavenger receptors, toll-like receptors and LOX-1 receptors, triggering cytotoxic, pro-inflammatory and apoptotic pathways in the cells [3,4]. Although oxLDL has mostly been studied in the context of atherosclerosis and cardiovascular disease, an increasing body of evidence points towards the involvement of oxLDL in metabolic syndrome, type 2 diabetes and cancer [5,6]. Altogether, these studies indicate that oxLDL is a link between metabolic dysfunction, oxidative stress and inflammation, and is thus a promising therapeutic target for patients with acquired and inherited lipid metabolic disturbances.

Previously, studies have shown that cardiovascular disease patients with higher anti-oxLDL IgM antibody levels have a reduced atherosclerotic burden and a better prognosis, suggesting that anti-oxLDL antibodies have protective characteristics in atherosclerosis [7,8,9]. Notably, antibodies targeting oxidized lipids and associated products, such as those present in oxLDL, are naturally produced from birth in mice and humans alike, even before the immune system encounters such threats [10]. These so-called “natural autoantibodies” consist of IgM antibodies that mainly bind to malondialdehyde (MDA) and to phosphorylcholine (PC), a head group commonly present in phospholipids. Whereas in healthy cells and native LDL the PC moiety of phospholipids is unavailable for recognition by the immune system, PC becomes exposed and available for antibody binding in oxidized lipids and apoptotic cells [11]. In addition, PC epitopes are found in Streptococcus pneumoniae, a bacterium well known for inducing respiratory and brain disease. As such, anti-PC IgM autoantibodies protect the host against oxidized self-antigens and foreign *S. pneumoniae* alike, thus acting as a primary line of defense against such pathogenic agents. Conversely, studies have shown that heat-inactivated *S. pneumoniae* immunization increases anti-oxLDL antibody levels and reduces disease progression and burden in animal models for atherosclerosis, non-alcoholic steatohepatitis and Niemann–Pick type C1 disease [11,12,13,14]. While the aforementioned diseases differ in etiology and affected tissues and organs, all are characterized by metabolic dysfunction, lysosomal cholesterol accumulation, oxidative stress and inflammation, indicating that anti-oxLDL antibodies may have a broad therapeutic application and may serve as a useful therapeutic strategy in a wide array of metabolic diseases.

In the current pilot study, our aim was to assess whether a pneumococcal vaccine increases anti-oxLDL and anti-PC IgM and/or IgG antibody levels in patients with inherited metabolic diseases. Patients with the following metabolic diseases were eligible for this study: familial partial LPD (OMIM entry # 151660), FH (OMIM entry #143890) and NP type B and C (NPB and NPC, OMIM entries #607616, and #257220 and #607625, respectively). Ultimately, four LPD, three FH and two NPB patients participated in this study. LPD is a rare genetic disease which results in abnormal adipose tissue distribution, predisposing patients to develop metabolic syndrome, type 2 diabetes mellitus, and cardiovascular and liver disease. On the other hand, FH patients possess genetic mutations that interfere with cellular uptake of LDL, leading to high blood LDL levels from childhood that put FH patients at risk of developing metabolic syndrome and metabolic syndrome associated diseases. Finally, NPB patients are characterized by deficits in sphingomyelinase activity which lead to lysosomal accumulation of sphingomyelin, cumulating in hepatosplenomegaly and lung disease. In order to determine whether pneumococcal vaccination increases anti-oxLDL and anti-PC IgM and IgG antibodies, patients received one active dose of Prevenar 13, a pneumococcal conjugate vaccine routinely used in the clinic. Plasma antibody levels were measured before and four weeks after vaccination. This study’s findings indicate that administration of a single dose of Prevenar 13 does not increase antibody levels against PC or oxLDL in metabolic patients. While studies with larger, more homogenous populations are required to confirm these findings, our study suggests that alternative therapeutical strategies are needed in order to target oxLDL in human patients.

## 2. Materials and Methods

### 2.1. Patient Recruitment and Inclusion

Participants were recruited at the outpatient clinic for metabolic diseases at Ziekenhuis Oost-Limburg (Genk, Belgium). Data were collected between April 2018 and March 2019. Patients with NPB and NPC disease, FH and familial partial LPD were eligible to participate in the study. Furthermore, in order to participate in the study (1) patients had to be older than 10 years of age; (2) patients must not present any health conditions that might interfere with the study procedures, including autoimmune diseases, immune deficiency, Hodgkin lymphoma and splenectomy syndrome; (3) patients must not consume alcohol in excess (>20 g/day for men and >10 g/day for women); and (4) patients must not be illiterate.

Eligible patients were informed about the study and invited to participate during regular appointments with their clinician. All study participants, or their legal guardians in the case of underage participants, signed an informed consent prior to the start of the study. The study was approved by the Ethics Committee at Leuven University and was registered under the EU Clinical Trials Register (EudraCT 2015-004846-25) and in the NIH clinical trials database (clinicaltrial.gov, study identifier NCT02707211). 

### 2.2. Study Procedure and Outcomes

Patients were randomly assigned identification codes during each visit, so that posterior sample analysis was blindly performed. On the first visit, fasted venous blood samples were taken, followed by a vaccination with the maximum recommended one-time dose of 0.5 mL of Prevenar 13 (Pfizer). The last visit took place after four weeks, during which blood samples were once again taken. During each visit, 20 mL of blood was taken via intravenous injection from each patient and stored in EDTA-coated tubes. Blood samples were kept in ice-filled insulated containers until they reached Maastricht University (Maastricht, the Netherlands) on the same day. At Maastricht University, blood samples were spun at 2700 g for 10 min at room temperature, and the resulting plasma was aliquoted and stored at −80 °C for posterior analysis. The planned outcome of this pilot study was to assess changes in lipid metabolism and inflammation following Prevenar 13 vaccination and subsequent increase in anti-oxLDL/PC antibody levels. Since we did not observe an increase in anti-oxLDL IgM antibody levels, our new goal was to assess anti-oxLDL and anti-PC antibody titers before, and four weeks after Prevenar 13 vaccination.

### 2.3. Antibody Measurements

Plasma levels of IgG and IgM antibodies against Prevenar, oxLDL (copper oxidized moiety) and PC-BSA were measured via chemiluminescent ELISA as previously described [15]. 

### 2.4. Statistical Analysis

Since some of the diseases included in this study are very rare (namely, LPD and Niemann–Pick type B (NP-B)) and this was a pilot clinical study, no power calculations were performed to calculate sample size prior to the beginning of the study. Primary study outcomes, namely, anti-oxLDL and anti-PC IgM and IgG antibody levels before and after Prevenar 13 vaccination, were measured via two-tailed paired *t*-test (* *p* ≤ 0.05; ** *p* < 0.01; *** *p* < 0.001; **** *p* < 0.0001). All data are expressed as the group mean ± standard deviation of the mean. Data were statistically analyzed using GraphPad Prism software (version 6, GraphPad Software Inc., San Diego, CA, USA).

## 3. Results

### 3.1. Patient Population and Study Outcomes

**Figure 1:**
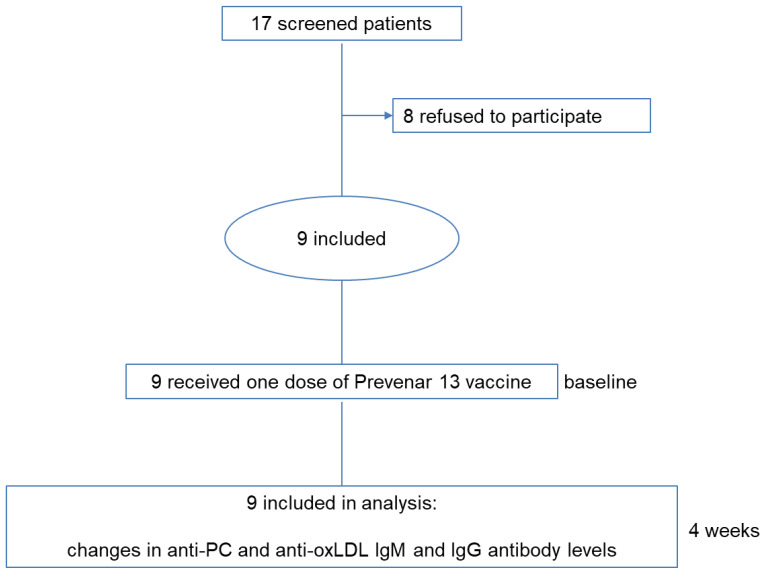
Study design.

Between 2017 and 2020, seventeen metabolic disease patients were approached to participate in this study, out of which nine were included (Figure 1). The final study population consisted of four familial partial LPD patients, three FH patients and two NPB patients. An overview of the study population is presented in Table 1.

### 3.2. Prevenar 13 Vaccination Induced an Increase in Prevenar-Specific IgM and IgG Antibodies

**Figure 2:**
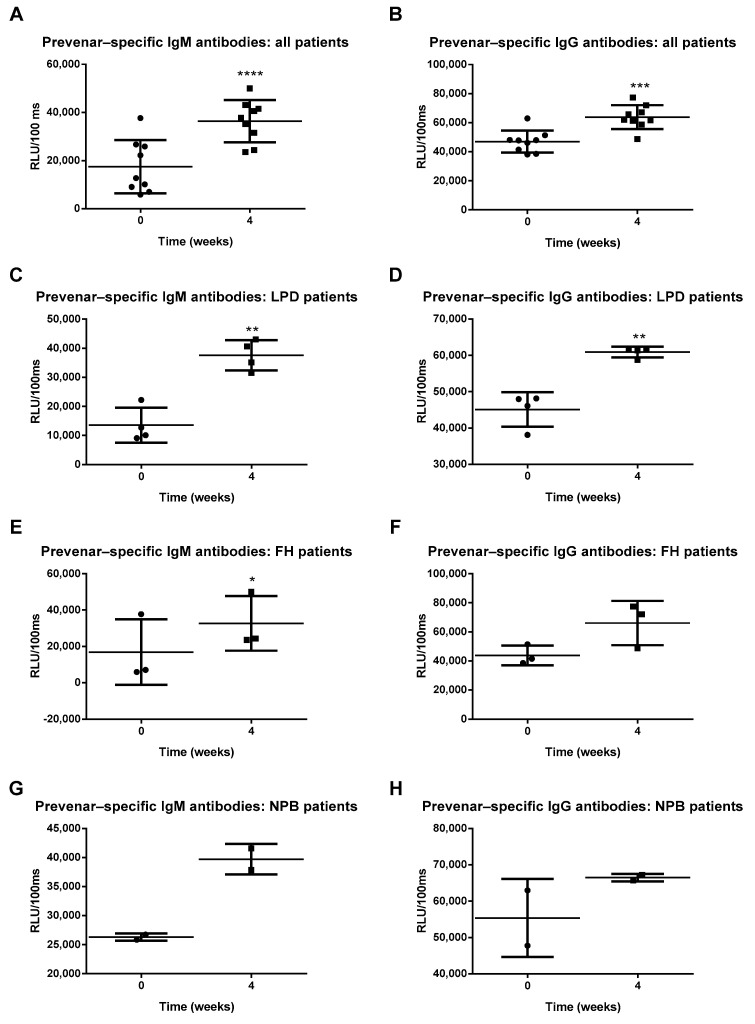
Prevenar-specific antibody titers. (**A**,**B**) Anti-Prevenar IgM and IgG antibody levels in all patients. (**C**,**D**) Anti-Prevenar IgM and IgG antibody levels in LPD patients. (**E**,**F**) Anti-Prevenar IgM and IgG antibody levels in FH patients. (**G**,**H**) Anti-Prevenar IgM and IgG antibody levels in NPB patients. * Indicates *p* < 0.05. All error bars represent SD.

In order to confirm that Prevenar 13 vaccination induced an immune response in patients, Prevenar 13-specific plasma IgM and IgG antibody levels were measured. Four weeks after vaccination, patients displayed increased anti-Prevenar 13 IgM and IgG antibody titers, proving that the vaccine induced an appropriate immune response (Figure 2A,B). To explore potential disease-specific differences within our study population, we further analyzed Prevenar-specific antibody levels among LPD, FH and NPB patients. In LPD patients, Prevenar 13 vaccination induced an increase in anti-Prevenar IgM and IgG antibodies (Figure 2C,D). Likewise, Prevenar 13 vaccination increased Prevenar-specific antibody levels in FH patients. Furthermore, we observed an increase in Prevenar-specific IgG antibody levels in FH patients, although this did not reach statistical significance (Figure 2E,F). While we could not perform statistical analysis on the data from NPB patients (*n* = 2), both anti-Prevenar IgM and IgG antibodies levels tended to increase following vaccination (Figure 2G,H). A complete overview of the descriptive statistical analyses is depicted in Table S1. Overall, these findings indicate that Prevenar 13 vaccination successfully elicited an immune response.

### 3.3. Prevenar 13 Vaccination Does Not Increase OxLDL or PC-Specific Antibody Titers

**Figure 3:**
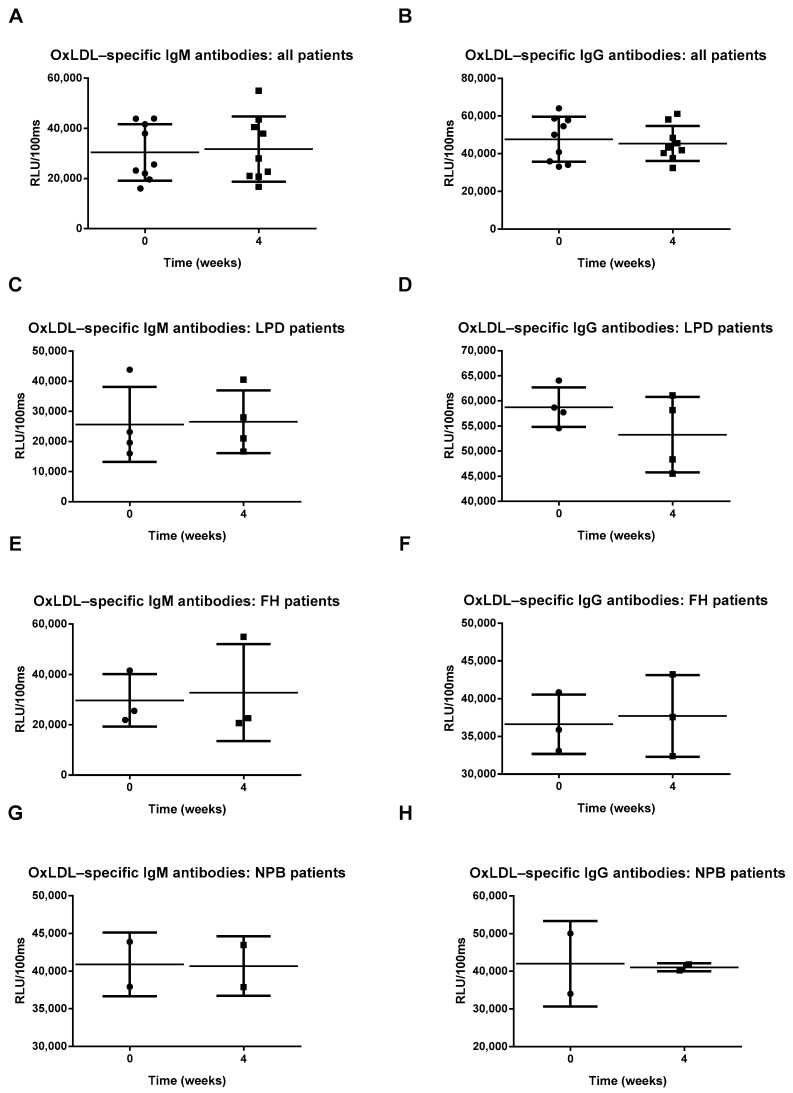
Oxidized low-density lipoprotein (OxLDL)-specific antibody titers (**A**–**H**). All error bars represent SD.

**Figure 4:**
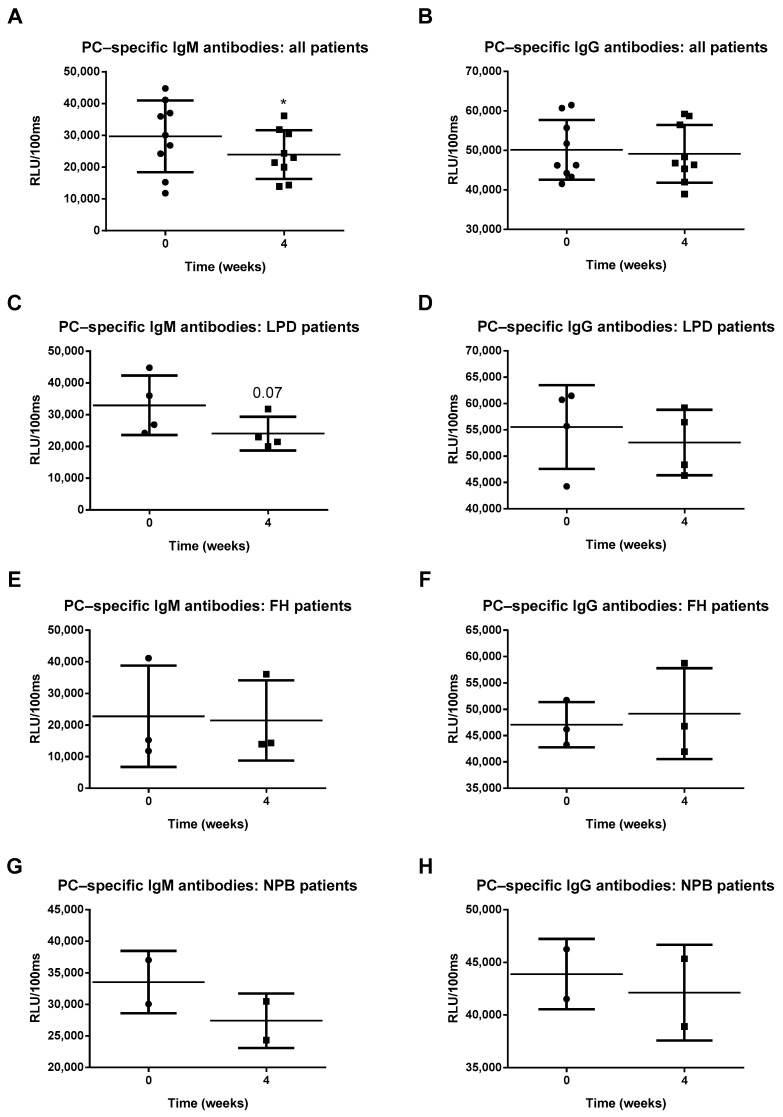
Phosphorylcholine (PC)-specific antibody titers (**A**–**H**). * Indicates *p* < 0.05. All error bars represent SD.

To determine whether Prevenar 13 vaccination induces an immune response against oxLDL, IgM and IgG antibody titers against oxLDL (copper oxidized moiety) were measured. No differences were observed in anti-oxLDL IgM and IgG antibody levels in either the overall patient population or the patient subgroups four weeks after Prevenar 13 vaccination (Figure 3A–H; Table S1), suggesting that the Prevenar 13 vaccine does not elicit an immune response against oxLDL in these groups. Finally, due to molecular mimicry between PC-moiety of S. pneumoniae and oxLDL, we analyzed the effect of Prevenar 13 vaccination on PC-specific IgM and IgG levels. Surprisingly, four weeks after receiving a single dose of Prevenar 13, we observed a reduction in anti-PC IgM antibody levels in all patients (Figure 4A). In contrast, no differences were observed in PC-specific IgG antibody levels in patients following Prevenar vaccination (Figure 4B). In LPD patients, Prevenar 13 vaccination tended to reduce anti-PC IgM antibody titers, although this effect did not reach statistical significance; no differences were observed in LPD patients following Prevenar 13 vaccination regarding anti-PC IgG antibody levels (Figure 4C,D). Regarding FH and NPB patients, Prevenar 13 did not elicit measurable changes in anti-PC IgM and IgG antibody levels (Figure 4E–H). More details on the statistical analysis are presented in Table S1. 

## 4. Discussion

In the current study, we analyzed whether a pneumococcal conjugate vaccine also elicits an immune response against oxLDL and/or PC in human metabolic disease patients. While patients displayed an appropriate IgM- and IgG-mediated immune response against the Prevenar 13 vaccine, Prevenar 13 vaccination did not increase antibody levels against oxLDL or PC in patients. These findings suggest that the Prevenar 13 vaccine is not a suitable tool to increase anti-oxLDL or PC antibodies in metabolic disease patients, and that alternative immunization strategies are needed in order to target oxLDL in humans. Further studies in larger, more homogenous populations should be performed to confirm our findings.

Oxidative stress, a phenomenon in which high amounts of reactive oxygen species overwhelm the cells’ antioxidant defenses, is a hallmark of metabolic syndrome, as well as a wide range of metabolic and/or inflammatory diseases. As a lipid-rich aggregate, LDL is prone to oxidative modifications, and thus is susceptible to oxLDL conversion under oxidative stress conditions. OxLDL, in turn, interferes with cell metabolism and activates pro-inflammatory and apoptotic pathways in cells, further impairing cellular metabolism and function, exacerbating the production of reactive oxygen species and triggering a vicious pathogenic cycle. Depending on the oxidative reagents and mechanisms, different portions of LDL can be modified to different extents; as such, it is likely that a heterogeneous population of oxLDL coexists in living organisms at any given time. Accordingly, antibodies targeting different oxLDL epitopes have been described in animals and humans alike, including antibodies against MDA or PC, other epitopes in copper-oxidized LDL, and against specific apoliprotein B peptides [16]. While several immunogenic groups are present in oxLDL, it is essential to establish the optimal oxLDL-related antigen for efficient immunization strategies, in order to avoid cross-reactions with non-pathogenic, non-oxLDL components. Being a common headgroup on phospholipids, PC is an abundant component of cell membranes and native LDL, but only becomes exposed and available as a so-called neo-antigen in apoptotic cells and oxLDL. In addition to being a neo-antigen, PC is a foreign antigen present on a variety of infectious agents, including *S. pneumoniae.* As such, among the different oxLDL immunogenic moieties, PC is an interesting candidate to explore, as (1) anti-PC autoantibodies have been found in human sera, (2) human anti-PC autoantibodies cross-react with oxLDL, and (3) PC is unlikely to induce an immune response against healthy cells or otherwise harmless cell components [17].

Previously, mice whose anti-oxLDL IgM antibody levels were increased by means of heat-inactivated *S. pneumoniae* displayed reduced disease burden in the context of NASH, NPC1 disease and atherosclerosis, strengthening the hypothesis that increasing anti-oxLDL antibody levels may be beneficial in humans. However, in contrast to observations in animal studies, in the current study, the Prevenar 13 vaccine did not increase anti-PC or anti-oxLDL antibody levels in metabolic disease patients. While we did observe a significant IgM and IgG-mediated response against Prevenar 13, it is possible that we could not measure a PC or oxLDL-specific immune response to the vaccine due to the limited sample size of the study and heterogenous composition regarding metabolic disease, sex and age of participants. In addition, it is possible that patients had received a pneumococcal vaccination in the past, which could impact their immune response against PC or oxLDL in the current study. Furthermore, it should be noted that, in the aforementioned in vivo studies, mice received an initial immunization consisting of heat-inactivated *S. pneumoniae* followed by several booster immunizations throughout the study period. In contrast, in this study, participants received a single dose of Prevenar 13 and were followed up for four weeks. As such, it is possible that a similar vaccination schedule is required to elicit an appropriate immune response against oxLDL and PC in humans. In addition, as already mentioned, mouse studies make use of heat-inactivated *S. pneumoniae* immunizations, in which the PC moiety is still present. In contrast, current pneumococcal vaccine manufacturing emphasizes the removal of PC-containing cell wall polysaccharides, as antibodies against these bacteria components are not considered protective in humans [18]. Therefore, available pneumococcal vaccines, including Prevenar, may not contain enough PC in their formulation to elicit an anti-PC and/or anti-oxLDL response. To address this issue, a study by Even et al. analyzed the effects of Prevenar vaccination on anti-PC antibody levels in apoliprotein E^−/−^ mice [19]. Following two Prevenar injections, the researchers observed an increase in anti-PC antibody levels, as well as reduced atherosclerotic burden in mice. While these results suggest that the Prevenar vaccination has enough residual PC in its composition to elicit an immune response, it is possible that vestigial PC amounts may differ between vaccine batches and therefore act as unreliable antigens [18]. Finally, it should be noted that key differences in both innate and adaptive immunity of mice and humans may lead to different immune responses following exposure to the same pathogen/epitope [20]. In addition, laboratory mice are kept in homogenous, aseptic and even sterile environments, which may also contribute to differences between immune responses of mice and humans to Prevenar 13 vaccination [21].

Previously, two studies reported that vaccination with a 23-valent polysaccharide vaccine (Pneumovax-23) has no effect on anti-MDA, anti-PC or anti- oxLDL in pediatric or adult patients [18,22]. While these findings appear to be in line with ours, it is worth mentioning that, while Prevenar 13 consists of 13 pneumococcal serotypes bound to a carrier that elicits a T-cell dependent immune response, Pneumovax-23 is a pneumococcal polysaccharide vaccine containing 23 *S. pneumoniae* serotypes whose immune response is T-cell independent. As such, Prevenar 13 elicits a stronger immune response than Pneumovax, particularly in young children and adults whose B-cells are not fully functioning [23]. Therefore, it is possible that Pneumovax-23 is not effective enough to elicit an appropriate anti-PC or anti-oxLDL response. Of note, a recent report by Grievink H.W. et al. found that two Prevenar 13 vaccinations, 28 weeks apart, increased anti-oxLDL and anti-PC IgG and IgM antibody levels in males, compared to participants who only received placebo injections [24]. It is possible that population size and heterogeneity, as well as the use of a single dose, did not allow the observation of a similar anti-oxLDL antibody increase following Prevenar 13 vaccination in the current study. Of note, the observed increase in anti-oxLDL antibody levels after two doses of Prevenar 13 in the discussed report reinforces the conclusion that Prevenar 13 composition retains molecular mimicry between *S. pneumoniae* and oxLDL, in line with a previously mentioned mouse study [19]. Whereas in mice, pneumococcal immunization leads to a strongly IgM-mediated immune response, in humans an IgG-mediated immune response against PC has been predominantly observed, further emphasizing the importance of species-related differences in the immune system [25]. As such, it is possible that a pneumococcal vaccine would elicit a strong anti-oxLDL IgG response, as was described in the aforementioned reports [24,25]. Of note, whereas anti-oxLDL IgM antibodies are widely considered protective, anti-oxLDL IgG antibodies have been reported as pro-atherogenic. As such, the previous report raises questions regarding the therapeutic potential of pneumococcal vaccination against metabolic and inflammatory diseases.

Overall, our study suggests that a single dose of Prevenar 13 does not elicit an increase in anti-oxLDL or anti-PC IgM antibodies in metabolic disease patients. While further studies are required to confirm our findings, due to the limited sample size and heterogeneity of patients included in this pilot study, these results emphasize the need to explore alternative strategies to increase oxLDL clearance. 

## Figures and Tables

**Table 1:** Characteristics of study population.

Disease	Sex	Age
Familial partial lipodystrophy	Female	28
	Female	38
	Female	37
	Female	38
Familial hypercholesterolemia	Male	15
	Female	11
	Male	13
Niemann–Pick type B	Male	36
	Male	38

## Data Availability

Data presented in this study can be requested to the corresponding author, Ronit-Shiri Sverdlov.

## Supplementary Materials

The following are available online at https://www.mdpi.com/2076-3921/10/1/129/s1, Table S1: Descriptive statistics.
