# Supplementary material for: The Influence of a Conjugated Pneumococcal Vaccination on Plasma Antibody Levels against Oxidized Low-Density Lipoprotein in Metabolic Disease Patients: A Single-Arm Pilot Clinical Trial
Source: Antioxidants (Basel). 2021 Jan 18;10(1):129. doi: 10.3390/antiox10010129 (PMC7831333; doi:10.3390/antiox10010129)
Supplement: Supplementary file 1 [file antioxidants-10-00129-s001.pdf]

Table S1 Descriptive statistics

	IgM			IgG		
	Difference mean (RLU/100 ms)	95% CI	P value	Difference mean (RLU/100 ms)	95% CI	P value
0-4 weeks (Prevenar-specific, all patients)	18934	14230 - 23637	< 0.0001	16921	9911 - 23930	0.0005
0-4 weeks (Prevenar-specific, LPD patients)	24057	16084 - 32031	0.0024	15853	10556 - 21151	0.0025
0-4 weeks (Prevenar-specific, FH patients)	15788	8379 - 23196	0.0117	22234	-9961 - 54430	0.0971
0-4 weeks (Prevenar-specific, NPB patients)	N/A	N/A	N/A	N/A	N/A	N/A
0-4 weeks (oxLDL-specific, all patients)	-2278	-7200 to 2645	0.3171	-2278	-7200 - 2645	0.3171
0-4 weeks (oxLDL-specific , LPD patients)	890.6	-7482 to 9263	0.7573	-5474	-14182 - 3233	0.1392
0-4 weeks (-oxLDL-specific , FH patients)	3053	-19161 - 25267	0.6142	1120	-2898 - 5138	0.3532
0-4 weeks (oxLDL-specific , NPB patients)	N/A	N/A	N/A	N/A	N/A	N/A
0-4 weeks (PC-specific, all patients)	-5746	-10094 - -1398	0.0159	-1015	-4945 - 2915	0.5679
0-4 weeks (PC-specific, LPD patients)	-8912	-19363 - 1540	0.0729	-2945	-12008 - 6117	0.3771
0-4 weeks (PC-specific, FH patients)	-1272	-10185 - 7641	0.6017	2061	-12202 - 16325	0.5975
0-4 weeks (PC-specific, NPB patients)	N/A	N/A	N/A	N/A	N/A	N/A
*All patients, N=9<br>LPD patients, N=4<br>FH patients, N=3<br>NPB patients, N=2						
